# Accelerating the K-Nearest Neighbors Filtering Algorithm to Optimize the Real-Time Classification of Human Brain Tumor in Hyperspectral Images

**DOI:** 10.3390/s18072314

**Published:** 2018-07-17

**Authors:** Giordana Florimbi, Himar Fabelo, Emanuele Torti, Raquel Lazcano, Daniel Madroñal, Samuel Ortega, Ruben Salvador, Francesco Leporati, Giovanni Danese, Abelardo Báez-Quevedo, Gustavo M. Callicó, Eduardo Juárez, César Sanz, Roberto Sarmiento

**Affiliations:** 1 Department of Electrical, Computer and Biomedical Engineering, University of Pavia, 27100 Pavia, Italy; emanuele.torti@unipv.it (E.T.); leporati@unipv.it (F.L.); gianni.danese@unipv.it (G.D.); 2 Institute for Applied Microelectronics (IUMA), University of Las Palmas de Gran Canaria (ULPGC), 35017 Las Palmas de Gran Canaria, Spain; sortega@iuma.ulpgc.es (S.O.); abaez@iuma.ulpgc.es (A.B.-Q.); gustavo@iuma.ulpgc.es (G.M.C.); roberto@iuma.ulpgc.es (R.S.); 3 Centre of Software Technologies and Multimedia Systems (CITSEM), Universidad Politécnica de Madrid (UPM), 28031 Madrid, Spain; raquel.lazcano@upm.es (R.L.); daniel.madronal@upm.es (D.M.); ruben.salvador@upm.es (R.S.); eduardo.juarez@upm.es (E.J.); cesar.sanz@upm.es (C.S.)

**Keywords:** K-nearest neighbors filtering, hyperspectral imaging instrumentation, brain cancer detection, image processing, graphics processing units

## Abstract

The use of hyperspectral imaging (HSI) in the medical field is an emerging approach to assist physicians in diagnostic or surgical guidance tasks. However, HSI data processing involves very high computational requirements due to the huge amount of information captured by the sensors. One of the stages with higher computational load is the K-Nearest Neighbors (KNN) filtering algorithm. The main goal of this study is to optimize and parallelize the KNN algorithm by exploiting the GPU technology to obtain real-time processing during brain cancer surgical procedures. This parallel version of the KNN performs the neighbor filtering of a classification map (obtained from a supervised classifier), evaluating the different classes simultaneously. The undertaken optimizations and the computational capabilities of the GPU device throw a speedup up to 66.18× when compared to a sequential implementation.

## 1. Introduction

Hyperspectral imaging (HSI) is a non-contact, non-ionizing and non-invasive imaging technique suitable for medical applications [1,2]. HSI combines traditional imaging and spectroscopy methods to obtain both spatial and spectral information of the captured scene [3]. HSI is becoming more popular in surgery applications as a guidance tool for surgeons, since it can provide more information than traditional imaging techniques, such as Magnetic Resonance (MR), Computed Tomography (CT), Ultrasound (US) and Positron Emission Tomography (PET), being a non-invasive and risk-free technique [4,5,6,7]. However, one of the main handicaps of this technology is the high computational requirements to process the large amount of data acquired by the sensor. The use of High Performance Computing (HPC) and highly parallelized algorithms is mandatory to achieve HSI intra-operative real-time processing [8]. 

The work presented in this paper is focused on the optimization, parallelization and implementation of the K-Nearest Neighbors (KNN) filtering algorithm on a Graphics Processing Unit (GPU) to obtain real-time performance. This work has been developed considering the results and intermediate data obtained during the deployment of the European HypErspectraL Imaging Cancer Detection (HELICoiD) FET project [9,10,11,12]. The goal of this project was to apply the HSI technique to discriminate between tumor and normal brain tissue during neurosurgical operations in real-time. The developed system provides surgeons with a guidance tool to assist them during brain tumor resections, avoiding both unintentionally leaving behind small remnants of tumors and the excessive extraction of normal tissue. This accurate delimitation of the tumor boundaries will improve surgery outcomes, therefore improving the patient’s quality of life.

The KNN algorithm is a classifier widely used in several research areas and also in the field of HSI, where a pixel-wise classification is performed [13]. The most relevant part of this method is the searching of the K nearest neighbors, which is a highly demanding task in terms of computational time. Since the main goal of most works in the state of the art is to execute this algorithm in real-time, or at least with reduced execution times, it becomes necessary to exploit the possibilities offered by high performance devices, being GPUs a highly appealing option. As a massively parallel architecture, this kind of devices has been widely used for exploiting data parallelism in several applications from different scientific fields [14,15,16], as well as in HSI [17,18].

Concerning the KNN algorithm, several parallel CUDA implementations have been proposed in the scientific literature. The results reported by these previous works point out that this technology is able to provide very high speedups compared to serial codes. For example, in [19], authors provide two CUDA versions of this algorithm, one characterized by custom kernels and the other exploiting the CUBLAS library (NVIDIA cuBLAS. Available online: https://docs.nvidia.com/cuda/cublas/index.html, accessed on 9 May 2018). Using synthetic data, they obtain speedups equal to 64× and 189×, respectively, compared to the highly optimized Approximate Nearest Neighbors (ANN) C++ library. They also apply the two parallel versions of the KNN algorithm to the high dimensional Scale-Invariant Feature Transform (SIFT) feature matching, obtaining speedups of 25× and 62×, respectively. In addition, in [20], the authors exploit the GPU technology to implement a new version of the KNN algorithm called *Sweet KNN*. This new algorithm is based on a Triangle Inequality (TI) approach, which tries to reduce the number of distance computations, since the goal of the work is to find a good balance between redundancy minimization and regularity preservation for various datasets. This work presents an average speedup of 11× compared to existing GPU implementations on KNN, with a maximum speedup of 120×. 

Recent uses of KNN algorithm show that it is not restricted to a classification role. In the last few years, it has also been used as a filtering technique to improve the results of spectral classifications by adding spatial domain information [21]. This work presents a parallel implementation of the KNN filtering algorithm, which can integrate the output of the Support Vector Machine (SVM) classifier with the one-band representation of a hyperspectral (HS) cube generated using the Principal Component Analysis (PCA) algorithm. The goal of the whole system is to perform real-time classification of brain cancer, where real-time restrictions are considered to be met when the processing time is lower than the time elapsed between two consecutive image acquisitions of the exposed brain (~1 min).

## 2. Materials and Methods

This section presents the HSI instrumentation employed to obtain the in-vivo HS brain cancer image database, the serial implementation of the algorithm as well as the optimizations and the parallelization analysis applied to the KNN filtering algorithm.

### 2.1. Hyperspectral Acquisition System

**Figure 1:**
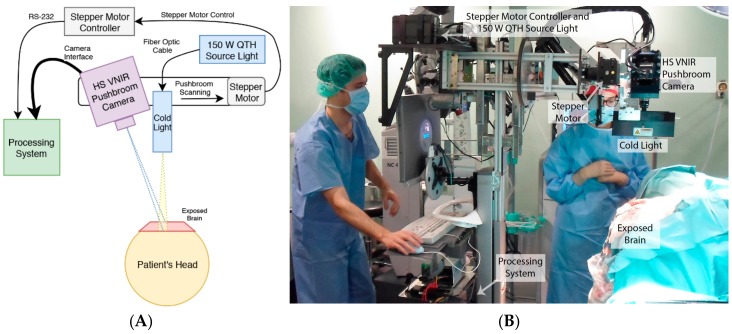
Hyperspectral acquisition system. (**A**) Schematic diagram of the HS acquisition system; (**B**) HS acquisition system capturing an image during a neurosurgical operation at the University Hospital Doctor Negrin of Las Palmas de Gran Canaria.

To obtain the HS in-vivo brain image dataset used in this study, a customized HS acquisition system was employed [12]. The acquisition system is composed by a Visual and Near Infra-Red (VNIR) *pushbroom* camera (Hyperspec^®^ VNIR A-Series, Headwall Photonics Inc., Fitchburg, MA, USA). This camera captures images within the spectral range between 400 and 1000 nm, obtaining 826 spectral bands with a spectral resolution of 2–3 nm and a pixel dispersion of 0.74 nm. In order to capture the complete HS cube, the camera uses a *pushbroom* scanning technique, which allows the 2-D detector to capture the complete spectral dimensions and one spatial dimension of the scene. By shifting the camera’s field of view with respect to the scene, the second spatial dimension is acquired. Figure 1A shows a schematic diagram of the acquisition system. The system is composed by an illumination device capable of emitting cold light in the range between 400 to 2200 nm. A Quartz Tungsten Halogen (QTH) lamp is connected to the cold light emitter through a fiber optic cable to avoid the high temperatures of the light in the exposed brain surface. In order to perform the *pushbroom* scanning, the system uses a stepper motor connected to the processing system via an RS-232 port. Figure 1B shows the HS acquisition system while capturing an HS image of the exposed brain surface during a surgical operation at the University Hospital Doctor Negrin of Las Palmas de Gran Canaria (Spain).

### 2.2. Hyperspectral Brain Cancer Image Database

In this study, a set of five in-vivo brain surface HS images have been employed to evaluate the performance of the KNN filtering implementation. These images were captured using the HS acquisition system, and they belong to adult patients undergoing craniotomy for resection of intra-axial brain tumor. Images were obtained at the University Hospital Doctor Negrin of Las Palmas de Gran Canaria (Spain) from four different patients with a confirmed grade IV glioblastoma tumor by histopathology. The study protocol and consent procedures were approved by the *Comité Ético de Investigación Clínica-Comité de Ética en la Investigación* (CEIC/CEI) of University Hospital Doctor Negrin and written informed consent was obtained from all subjects.

HS images were obtained intra-operatively after craniotomy and resection of the dura. Before the images were captured, the operating surgeon initially identified the approximate location of normal brain and tumor. Rubber ring markers were then placed on these locations and the images were taken with the markers in situ. At that point, tissue samples were resected from the marked areas and sent to pathology for tissue diagnosis. Depending on the location of the tumor, images were acquired at various stages of the operation. In the cases with superficial tumors, some images were obtained immediately after the dura was removed, while in the cases with deep laying tumors, images were obtained during the actual tumor resection.

The selected HS images were pre-processed following the pre-processing chain presented in [12]. This chain is composed by four steps: image calibration, noise filtering, band averaging and pixel normalization. In the first step, after the image acquisition, the HS raw data are calibrated using a white reference image (captured from a certified white reference tile in the same illumination conditions in which the images were captured) and a dark reference image (obtained by keeping the camera shutter closed). This calibration is performed to avoid the problem of the spectral non-uniformity of the illumination device and the dark currents of the camera sensor. Next, due to the high spectral noise generated by the camera sensor, a set of steps are applied to remove this noise from the spectral signatures and to reduce the number of bands of the samples without losing the main spectral information. Finally, a normalization step is performed in order to homogenize the spectral signatures in terms of the reflectance level. The final HS cube is formed by 128 spectral bands, covering the range between 450 and 900 nm [12].

**Figure 2:**
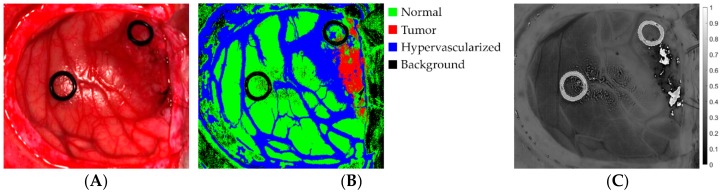
Example of an in-vivo HS human brain image dataset employed in the study (P2C1). (**A**) Synthetic RGB representation of the HS cube; (**B**) Supervised classification map obtained using the SVM classifier; (**C**) One-band representation of the HS cube obtained employing PCA algorithm.

Figure 2A shows an example of the synthetic RGB representation of an HS cube from the HS brain cancer image database used in this study. Furthermore, each one of these images was processed using a supervised SVM classifier (Figure 2B), while a one-band representation was obtained using PCA algorithm (Figure 2C). Table 1 details the characteristics of each HS image, where PXCY stands for Patient X and Capture Y.

Four different classes were labeled in the images for the supervised classification: tumor tissue, normal tissue, hypervascularized tissue (mainly blood vessels) and background (other materials or substances that can be presented in the surgical scene that are not relevant for the tumor resection process). These classes were represented in the classification maps with the following colors: red, green, blue and black, respectively.

### 2.3. K-Nearest Neighbors Filtering Algorithm

**Figure 3:**
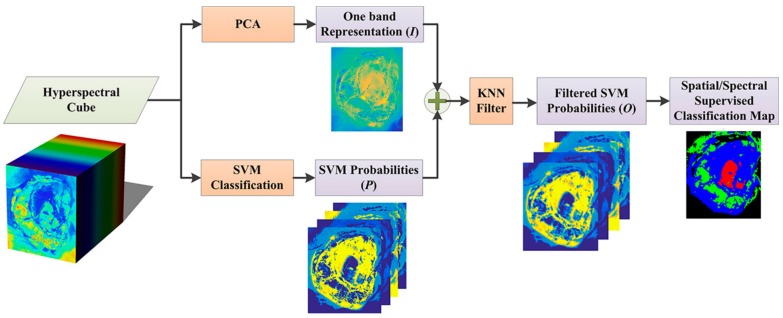
Block diagram of the KNN based spatial-spectral classification.

As introduced in Section 1, the KNN algorithm has recently been widely used in the field of HSI as a filtering technique [21] to refine outputs from classifiers, in this case a SVM, with the spectral information computed trough the PCA algorithm. As shown in Figure 3, the KNN-based filtering algorithm [21,22] receives an input image *P*, which is composed of the probability maps estimated by the SVM classifier and a guidance image *I*, i.e., the one-band representation of the HS cube, generated using a dimensional reduction algorithm, such as PCA. The output of this algorithm is a filtered classification map based on the highest probability assigned to each pixel in the previous classification stage [21].

In this method, the nearest neighbors of a certain pixel are searched in a feature space, which contains both the pixel value and the spatial coordinates. This space is defined by a feature vector *F*, as shown in Equation (1):

(1) F(q)=(I(q),λ·l(q), λ·h(q)) 

where *I*(*q*) is the normalized pixel value of the guidance image and *l*(*q*) and *h*(*q*) refer to the normalized coordinates of pixel *q*. The spectral value of the pixel and its spatial coordinates are weighted with *λ*, which is a balance parameter to weigh the importance of the spatial information in the neighbors searching: if *λ* is zero, the spatial information will not be considered; if its value is higher than zero, more influence is given to the local neighborhood in the filtering process [21,22].

The neighbors searching requires the computation of the distances between pixels on the base of the data contained in the feature vector. The distance from a given pixel located at (*r*, *c*) coordinates of the image to any other pixel at (*i*, *j*) can be computed using the Euclidean distance, i.e., the 2-norm:

(2) $$d\left(I\left(rc\right),\;I\left(ij\right)\right)=\sqrt{{\left(I_{rc}-I_{ij}\right)}^{2}+{\left(r-i\right)}^{2}+{\left(c-j\right)}^{2}}\;$$

where $I_{rc}$ is the normalized pixel value of the guidance image *I* at row *r* and column *c* and $I_{ij}$ is the value of every other pixel at row *i* and column *j*.

In this work, also the Manhattan metric (Equation (3)) has been used to compute the distances, considering always all the data contained in the feature vector. In Section 3, a comparison between the implementations performed using these two metrics will be presented, underlining both the differences in terms of processing time and classification results:

(3) $$d\left(I\left(rc\right),\;I\left(ij\right)\right)=\left|I_{rc}-I_{ij}\right|+\left|r-i\right|+\left|c-j\right|\;$$

Once the distances for each pixel are computed, the algorithm has to sort them to select the K nearest neighbors. After the KNN searching is concluded, the algorithm continues with the filtering step, whose output is the optimized probability *O*(*q*). For each pixel, it computes a number of outputs equal to the number of SVM classes. In particular, for each pixel *q* and each SVM class, it computes the optimized probability *O*(*q*), defined as follows:

(4) $$O\left(q\right)=\frac{\sum P\left(s\right)}{K},s\in \omega_{q}\;$$

where *P* is the original probability map (one per class) generated by the SVM classifier, $\omega_{q}$ indicates the set of *K* nearest neighbors of the pixel *q* and *s* is the index related to each neighbor of the previous set [22].

The last step of the algorithm consists of assigning a label to each pixel to generate a new final classification map. The label that is assigned to each pixel of the image is the class with the highest optimized probability.

## 3. KNN Filtering Algorithm Implementation

After describing all the steps of the KNN algorithm, this section will introduce the optimizations and the parallelization analysis performed to the algorithm in order to reduce its computational cost.

### 3.1. KNN Search Optimization

After an extensive analysis of the computational cost of the KNN filtering algorithm, it is possible to determine that the neighbors searching phase is the most consuming part of the code. For this reason, the first optimization proposed is the definition of a *search window* in the neighbors’ selection. This *search window* is a region close enough to the pixel whose neighbors are going to be chosen. In the original algorithm, this step consisted on computing, for each pixel, a number of distances equal to *Npixels* − 1, where *Npixels* is the number of pixels in the image. Our approach is to search the *K* nearest neighbors of a pixel within this window, not considering the entire image, in order to reduce the number of computed distances, as the probability to find smaller distances in further zones of the image tends to 0.

Concerning the parameter setting, in [22] it is asserted that λ = 1 and *K* = 40 are a good compromise for this medical imaging application. This value of λ gives a high importance to the spatial information, in particular to the local neighborhood. Considering this value, and the fact that the values of the guidance image *I* (i.e., the first term in Equation (2)) are normalized to 1, it is easy to foresee the behavior of the sorting algorithm in the neighbors’ selection. This allows introducing heuristic considerations that will help reducing the execution time. For any given pixel $I_{rc}$ in the image, the distance computation will follow a pattern determined by the spatial distance, i.e., the last two terms in Equation (2), which are related to the spatial coordinates of the pixel. These two values will hence dominate the equation once they overcome the spectral value (i.e., the first term in Equation (2)), since they will contribute to a distance value for any other pixel that will be always predominant if such pixels are far enough from the location of the pixel under consideration. In other words, it is a sufficient condition to sort only a certain subset of pixels in a region close enough to the pixel whose neighbors are being searched. This effectively reduces the search space and the computational cost.

Section 4 compares the computational time and classification results for both the serial and the parallel implementations varying the window sizes (*WSize*). After several analyses, a window with *WSize* = 14, i.e., 14 rows of the image, has been selected, so the search space contains a number of pixels equal to *14 rows* × *total number of columns*. This choice has been taken after evaluating different window sizes (ranging from *WSize* = 20 to *WSize* = 2) in order to find which one guarantees the same classification results of the version characterized by the entire image as the searching space. The *WSize* ≥ 14 presents the same classification results than the use of the entire image. A row-wise window has been selected instead of a column-wise one in order to have all the data stored sequentially. The version characterized by this window size has been chosen as the *reference result* because the classification results are the same compared to the implementation that considers the entire window.

**Figure 4:**
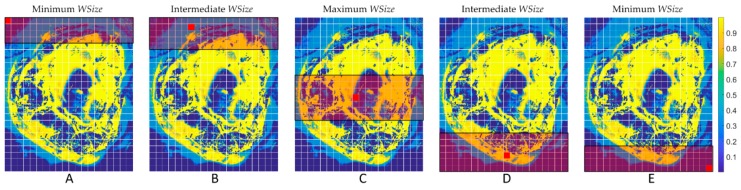
KNN window searching method example. (**A**) Minimum window size of the first pixel; (**B**) Intermediate window size of a pixel near the upper border; (**C**) Maximum window size of a pixel in the center of the image; (**D**) Intermediate window size of a pixel near the bottom border; (**E**) Minimum window size of the last pixel.

The window is considered in a symmetric way with respect to the pixel that is being processed, so one half of the window is evaluated above the pixel and the other half below it (Figure 4). In order to avoid the effect of the borders, those pixels near them are treated separately. In this case, to maintain a certain spatial coherence, the size of the window for the pixels in the top-most rows is smaller at first, so as not to search further than *WSize/2* down in the image. In this way, the band grows with each further pixel being processed until the steady state is reached. This happens when the number of pixels above the one being processed reaches *WSize/2* and it is kept until an analogous situation happens in the lower zone of the image.

### 3.2. Serial Implementation

**Figure 5:**
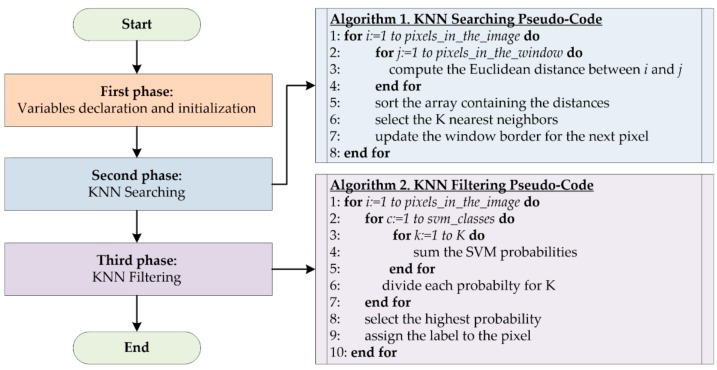
Flow diagram of the serial implementation of the KNN filtering algorithm.

The serial implementation of KNN algorithm is written in C language and presents three main phases, as shown in Figure 5. The first one concerns the declaration and initialization of all the variables, arrays and structures needed in the computation. For example, for each pixel, two types of structures are defined: the former, *featureMatrixNode*, contains all the parameters needed in the computation of the Euclidean distance, shown in Equation (2); the latter, *featureDistance*, contains the distances (between the considered pixel and the pixels within its window) and the indexes of these pixels.

The second phase refers to the *K* nearest neighbors searching. Considering every pixel in the image (Algorithm 1, line 1), the algorithm computes the distances between it and all the pixels inside its window (Algorithm 1, lines 2–4), exploiting the Euclidean metric in Equation (2). After storing all the distances in the *featureDistance* structure, the algorithm sorts them in ascending order through the Merge Sort algorithm (Algorithm 1, line 5) and selects the indexes of the *K* pixels with lowest distances (Algorithm 1, line 6). At the end of this phase, the parameters related to the window sizes are updated (Algorithm 1, line 7) on the base of the pixel location, as described in Section 3.1.

Once all the neighbors of each pixel have been computed, the KNN filtering phase starts (Figure 5, Algorithm 2). Its goal is to assign a label to each pixel considering the probability maps generated by the SVM algorithm. In this phase, the algorithm computes, for each pixel, a number of optimized probabilities *O*(*q*), described in Equation (4), equal to the number of the SVM classes, which are four in this work. In particular, for each class, the SVM probabilities of all the neighbors of the pixel that is going to be processed are added (Algorithm 2, lines 1–5). Then, the result is divided by the number of neighbors (*K*) (Algorithm 2, line 6). After computing the four optimized probabilities *O*(*q*) for each pixel, the algorithm selects the highest value and assigns the label of the corresponding class to the pixel (Algorithm 2, lines 8–9). The code was compiled using Microsoft vc140 compiler, enabling advanced optimizations using the Release compilation profile and flags such as -O3.

### 3.3. Parallel Implementation

**Figure 6:**
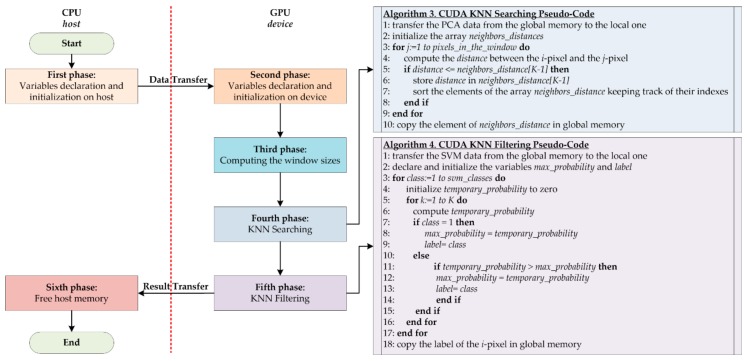
Flow diagram of the parallel implementation of the KNN filtering algorithm.

A parallel version of the KNN filtering algorithm has been developed in CUDA to exploit the NVIDIA GPU technology. The GPU device used during the tests was an NVIDIA Tesla K40 GPU (NVIDIA TESLA K40 GPU Specifications. Available online: https://www.nvidia.com/content/PDF/kepler/Tesla-K40-Active-Board-Spec-BD-06949-001_v03.pdf, accessed on 9 May 2018). This board is based on the Kepler architecture (working at 875 MHz) and it contains 2880 CUDA cores and 12 GB GDDR5 memory with a peak bandwidth of 288 GB/s. The board is connected to the CPU host through a PCI Express 2.0. The basic idea followed in this approach is that each CUDA core has to assign a label to each pixel in parallel. Figure 6 shows the main phases of the parallel implementation. The flow starts on the host with the declaration and initialization of all the variables (*First phase* in Figure 6). The main difference between this first phase and the corresponding one of the serial code is that, in this parallel implementation, the number of arrays, structures and variables allocations is decreased in order to save memory.

After the first phase, the algorithm transfers to the device the guidance image I generated by the PCA algorithm and the probability maps generated by the SVM classifier. The flow proceeds with the resources allocation on the device (*Second phase* in Figure 6). The first step of the KNN filtering algorithm on the GPU device concerns the execution of a kernel that evaluates the borders and the size of the windows in parallel through the pixels (*Third phase* in Figure 6). Contrary to the serial code execution, where the parameters related to the window dimensions are updated at the end of the neighbors’ selection for each pixel, in the parallel version the algorithm needs to know these variables before starting the KNN filtering computation. In fact, in the following steps, it is important to copy the PCA and SVM data (already transferred to the device) from the global to the local memory of the GPU, shared by the threads within a block. For this reason, each thread copies the part of the data (delimited by the window parameters) needed in the computation. Then, the results are copied to the global memory only at the end of the kernel execution. This step is crucial to decrease the execution time since the accesses to the global memory are very slow. 

In the fourth phase, which corresponds to the second phase of the serial code, each thread evaluates the *K* nearest neighbors of a pixel in parallel (Figure 6, Algorithm 3). First, the PCA data required by each block are copied from global to the local memory (Algorithm 3, line 1). Then, each thread of the block declares an array called *neighbors_distances*, whose dimension is equal to the number of *K* neighbors, which is set to 40 in this work. This array is initialized with large values and will contain the 40 lowest distances computed between the pixels (Algorithm 3, line 2). The implementation goes on computing the distance between pixel *i*, represented by the thread, and all the pixels within its window (Algorithm 3, lines 3–4). If the distance between the pixels *i* and *j* is smaller or equal to the last element of the *neighbors_distances* array, this distance will be stored in the last position of the array (Algorithm 3, lines 5–6). It is important to highlight that this *if condition* is always verified considering the first 40 pixels in the window (i.e., the first 40 *for* loop iterations). Once the first 40 iterations are executed, in the last position of the array there will be a *real* distance (not the initialization value) and it will be the highest value among those already present in the array. This is verified because every time that a new distance is stored in the array, the algorithm calls a *sort function* in order to sort the elements of the array in an ascending order, keeping track of their indexes (Algorithm 3, line 7). The *K* indexes of the selected neighbors are the output of the kernel and will be copied to the global memory (Algorithm 3, line 10).

**Figure 7:**
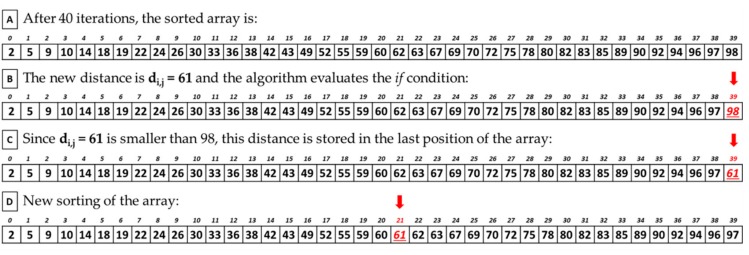
KNN searching new distance evaluation example.

Figure 7 shows an example of the evaluation of a new distance by the KNN searching algorithm. After the first 40 iterations, the array contains 40 distances stored in ascending order (Figure 7A). When a new distance is computed (in this example its value is 61), it is compared with the last element of the array, in this case located in position 39 and whose value is 98 (Figure 7B). Since the new distance is lower than 98, it is stored in the last position of the array (Figure 7C). At this point, the array is sorted again (Figure 7D). Due to the reduced dimension of the array to be sorted, the sort function implemented in this work is the *shell sort* algorithm.

After computing all the neighbors for all the pixels, the fifth step of the KNN algorithm starts. In this phase, the KNN filtering is computed by every thread of each block (Figure 6, Algorithm 4). First, each thread copies the SVM probabilities of their corresponding neighbors from the global to the local memory (Algorithm 4, line 1). For each class, the algorithm computes the *temporary_probability* value of each pixel, which is the sum of the SVM probabilities of all the neighbors of the reference pixel (Algorithm 4, lines 3–6). If the algorithm is executing the first iteration of the first *for* loop (i.e., if it is considering the first class), the variable *max_probability* assumes the value of *temporary_probability* variable and the index of the class is stored in *label* (Algorithm 4, lines 7–10). In the following iterations, after computing the *temporary_probability*, its value is stored only if it is higher than the *max_probability value* (which represents the highest probability value of the previous classes). In this case, the index of the class is also stored (Algorithm 4, lines 11–14). At the end of the *for* loop that iterates on the number of classes, the algorithm selects the label of the pixel corresponding to the highest sum of probabilities among the four classes. It is worth noting that the algorithm immediately evaluates if the sum of probabilities could be the highest among the classes or not. This fact means that some arrays declared in the serial version can be replaced with a few variables, thus saving memory. At the end of this phase, the label of the pixel, which is the output of this step, is stored in the global memory (Algorithm 4, line 18). It is worth noting that, for each kernel, the block dimension is equal to 32. This choice is related to the *warp* dimension defined by the CUDA framework, since the GPU scheduler considers 32 threads as the fundamental scheduling unit.

Once the KNN algorithm execution ends on the GPU device, an array containing the labels of all the pixels is transferred from the GPU device to the CPU host. At this point, the memory can be released (*Sixth phase* in Figure 6).

## 4. Experimental Results and Discussion

This section presents the results of the implementations of the KNN-based filtering algorithm by evaluating different sets of parameters, analyzing both the computational times and the classification accuracy.

### 4.1. KNN Window Search Optimization Results with Euclidean Distance

Section 3.1 described an important optimization introduced in the serial and parallel implementations concerning the computation of the distances between pixels inside a window and not within the entire image. Reducing the space where the algorithm evaluates the distances ensures a significant decrease of the computational time, as shown in Table 2. In particular, the table provides the execution times for all the images, considering both the case in which the neighbors are searched within the entire image (*EI*) and within a window of 14 rows (*WSize14*). The speedup obtained with the optimization has been also included. In addition, this table shows the total number of pixels of each image and the number of pixels inside the smallest and the biggest window in the *WSize14* implementation. The times refer to tests where the Euclidean distance has been considered. The simulations of the serial code have been carried out on an Intel i7 processor, working at 3.50 GHz, equipped with 16 GB RAM. The execution times shown in the following tables have been measured using the clock routine included in the *time.h* header file. 

Data presented in Table 2 show that this optimization allows a huge decrease in the execution times. For example, considering the biggest image of the dataset, P1C2, the time of the implementation for the entire image is 19,135.58 s (about 5 h and 30 min). On the contrary, considering a window of 14 rows, this time decreases to 509.16 s (about 8 min). The reason of this huge time difference is that, when the algorithm has to consider the entire image, it needs to compute a number of distances equal to (264,408–1) for each pixel, where 264,408 is the number of pixels of the P1C2 image. However, with the window technique, the algorithm computes a number of distances that, for the same image, varies from (3864–1) to (7728–1), where 3864 and 7728 are the number of pixels inside the windows with the minimum and the maximum sizes, respectively (depending whether the pixel is in the borders or in the center of the image). It is important to highlight that the highest speedup is achieved analyzing the image P2C1, since it has a number of pixel which is an integer multiple of 2, allowing better use of the CPU resources and faster memory accesses. The other images have not this characteristic and thus the CPU execution is not so optimal. Concerning the classification results, it is important to notice that there are no differences in the results, and therefore all the pixels are classified with the same labels, using either the entire image or a window.

Considering this significant result, the computational time variations were evaluated when the window size was reduced. Furthermore, since the main goal of the work was to reach real-time execution, a parallel version of the algorithm was developed in CUDA language to exploit the GPU technology. Table 3 shows the execution times of the serial and parallel implementations characterized by window sizes that vary from 14 to 2 with decrements of 2. In addition, the speedups between the serial and the parallel codes (executed onto the Tesla K40 GPU) are presented.

The reduction of the window size supposes a decrease in the execution times because the algorithm has to compute a lower number of distances. For example, considering the P1C2 image, the time varies from 509.16 s (~8 min) to 62.36 s (~1 min) in the serial versions of *WSize14* and *WSize2*, respectively. If the parallel implementation of the same image is considered, the times present a further decrease. In fact, for the same image the parallel version of *WSize14* is ~37× times faster than the serial version, taking only 13.53 s instead of ~8 min. At the same time, the parallel execution of *WSize2* takes only 1.26 s instead of ~1 min (the speedup is ~49×). Concerning all the images in the reference implementation *WSize14*, the speedups are always higher than 35× and, in the best case (P4C1), it reaches 43×. If we consider all the other versions, with the decreased windows sizes, the parallel code shows even higher speedups. For example, considering the P1C2 image and the window size *WSize10*, the parallel code takes 6.48 s while the serial version takes 408.52 s (~6 min), obtaining a speedup of ~63×. Nevertheless, it is necessary to examine these times and speedups also taking into account the classification results. It is very important to consider if, when reducing the window size, there are pixels classified with different labels compared to the *reference* version (*WSize14*). Table 4 shows the number of misclassified pixels between the reference result and other window sizes. Additionally, the percentage of the difference is shown.

**Figure 8:**
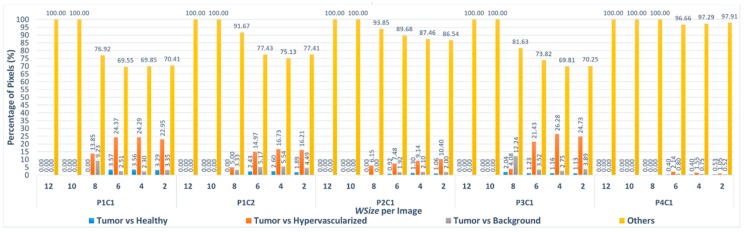
Percentage of pixels that have been misclassified using the Euclidean distance between tumor and healthy tissues (blue), tumor and hypervascularized tissues (orange), tumor tissue and background (gray) and the other misclassifications between healthy, hypervascularized and background (yellow). The results were obtained per each window size implementations compared to the *WSize14* for each image of the dataset.

Considering the first three windows sizes (*WSize12*, *WSize10*, *WSize8*) for all the images, the number of pixels classified with different labels is very low, taking into account the final application of the system. In fact, the highest percentage of different pixels is 0.057%, and it is related to the P4C1 image, which, in the *WSize8* version, presents 71 different pixels on a total amount of 124,691 pixels. Concerning the other three windows sizes, the highest percentage of different pixels for window *WSize6* is 1.46% considering the P1C1 image (3672 different pixels on 251,532). For the window *WSize4*, the percentage of different pixels is ~3.62%, referred also to the P1C1 image (9096 different pixels on 251,532) and for *WSize2*, the highest percentage is ~8.341%, considering the biggest image of the database, P1C2 (22,054 different pixels on 264,408). At this point, there is a further evaluation that can be made considering that this algorithm is part of a system whose main goal is to discriminate between tumor and healthy tissue. The classification is made between four classes that are normal tissue, tumor tissue, hypervascularized tissue and background [22]. From the surgical and medical point of view, it is clear that a wrong discrimination between tumor and healthy tissue has much greater and transcendental relevance than just a misclassification issue between tumor and any other classes (hypervascularized and background) or between healthy, hypervascularized and background classes. It is possible to re-evaluate again the results of Table 4, considering that, in the different *WSize* executions, only a low percentage of different pixel labels are exchanged between tumor and normal tissue. Figure 8 shows the percentage of pixels that are misclassified between tumor and healthy tissues, tumor and hypervascularized tissues and tumor and background, respectively, considering all the windows sizes for each image compared to the reference version. In addition, the graph presents the classification differences between healthy, hypervascularized and background classes (called *Others*).

As it can be seen in Figure 8, only in the case of the P3C1 image using the *WSize8*, the algorithm misclassifies approximately 2% of the pixels (1 out of 49 pixels), exchanging the labels between tumor and healthy tissues. In all the other implementations of *WSize8*, the classification differences do not involve the tumor class. Furthermore, in the versions related to the three smallest windows (*WSize6*, *WSize4*, *WSize2*), the percentage of the pixels exchanged between these two classes is lower than the percentages of pixels exchanged between the other classes. For example, for the biggest image of the database (P1C2), in the *WSize6* implementation, the classification difference between tumor and healthy tissue represents 2.43% out of 2845 different pixels. Considering the same image in the *WSize4* and in the *WSize2* implementations, this percentage is 2.60% out of 7705 pixels and 1.89% out of 22,054 pixels, respectively. The highest percentage of differences between these two classes is found in the *WSize6* version regarding the P1C1 image, where it is around 3.57% out of 3672 pixels. According to these data, it is clear that the algorithm can correctly distinguish the tumor from the healthy tissue, while it makes more errors in separating the tumor from the hypervascularized tissue. The highest percentages of misclassified pixels between the tumor and the hypervascularized classes reach 26.28% of the total number of different pixels (P3C1 image, *WSize4* version). In fact, according to what it is said in [22], these two classes referred to tissues with similar spectral signatures that can produce some misclassifications. On the other hand, the spectral signatures of tumor and healthy tissues present remarkable differences that allow the algorithm to distinguish these two classes in the classification. 

### 4.2. KNN Window Search Optimization Results Using Manhattan Distance

As it was said before, the neighbors search represents the heaviest computational load of the KNN filtering algorithm. Although the distances computation is the most time-consuming task, the number of evaluated distances has been reduced in this study by considering a window, so a smallest area is considered instead of the entire image. To further reduce the execution time of this phase, the Manhattan metric has been tested instead of the Euclidean one, as described in Equation (3). Table 5 compares the times of the serial code using both the entire image (*EI*) and the reference window (*WSize14*), applying both the Euclidean and the Manhattan distances. The speedup obtained using the Manhattan distance and the percentages of pixels that are different in the results are also presented in this table.

As said in the previous paragraph, searching the neighbors within a window instead of in the entire image allows saving time without changing the classification results. A further reduction of the execution time is obtained using the Manhattan metric in the distance computations. In fact, for the biggest image of the database (P1C2), the time is reduced from ~5 h (19,135.58 s) using the Euclidean distance to ~2 h (7683.44 s) in the case of using the entire image. If the neighbors are searched within the window (for the same image), the time decreases to ~3 min (202.42 s) using the Manhattan distance. Concerning all the images, it is possible to reach speedups from 2.22× to 3.33×, considering the versions with the entire image, and from 2.49× to 2.66× in the *WSize14* executions. Comparing the implementations that exploit the Manhattan distance and the ones that use the Euclidean metric, the number of pixels classified with different labels is quite low: the highest percentage of different pixels is 1.33% in the P1C1 image. Furthermore, it is important to highlight that there are no differences in the classification results comparing the entire image and the *WSize14* versions, using the Manhattan distance.

At this point, it is interesting to evaluate how the execution times can be reduced changing the size of the windows using the Manhattan metric in the distances computation. The results shown in Table 6 confirm that decreasing the number of distance computations, i.e., the variations of the window sizes, allows further reductions of the computational time. The lowest execution times are obtained exploiting the GPU technology that can run the parallel algorithm taking ~8 s (compared to ~3 min) if the biggest image (P1C2) with the *WSize14* version is considered. The speedups obtained using this device and the optimizations introduced in the code are significant and they can reach up to 33.2× (P2C1-*WSize12*). For some images and for some window dimensions, the algorithm takes only a few seconds, but what is even more important to consider is the number of pixels that are misclassified when the window size decreases (Table 7).

As it can be seen in the results shown in Table 7, *WSizes12* and *WSizes10* present a reduced number of different pixels compared to the other implementations. Analyzing the Euclidean distance results presented in Table 4, this consideration can be made for the first three tests (*WSizes12*, *WSizes10* and *WSize8*) but, in this case, the number of different pixels in *WSize8* is higher than the first two versions. Despite this, it is important to highlight that the classification differences shown in Table 7 are not very relevant for the final application of the system. In this application, a solution with a good compromise between real-time execution and classification accuracy of the results has to be selected. In addition, it is also important to evaluate the percentage of different pixels that are misclassified between tumor and healthy tissues and between tumor and the other classes.

**Figure 9:**
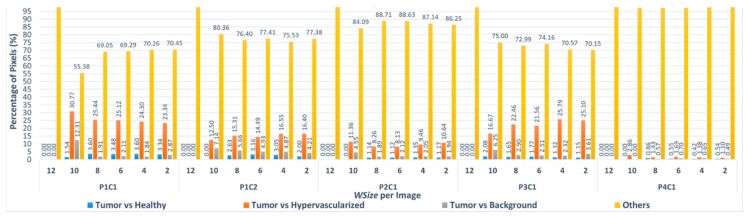
Percentage of misclassified pixels using the Manhattan distance between tumor and healthy tissues (blue), tumor and hypervascularized tissues (orange), tumor tissue and background (gray) and the other misclassifications between healthy, hypervascularized and background (yellow). The results were obtained per different window sizes implementation compared to the *WSize14* for each image of the dataset.

Figure 9 shows the percentage of pixels that are misclassified using the Manhattan metric between the different classes. In this figure, it is possible to notice that the algorithm misclassifies more pixels between tumor and hypervascularized classes than between tumor and healthy classes. In fact, the highest percentage of pixels misclassified is 30.77% related to the P1C1 image with *WSize10*, where the algorithm exchanges the labels of 20 pixels (between tumor and hypervascularized tissue) out of a total amount of 65 different pixels compared to the reference version *WSize14* (Table 7). Concerning the comparison between tumor and healthy classes, the number of pixels with an exchanged label is very low: the worst case is always the P1C1 image (*WSize8*), where 66 out of 1832 different pixels are misclassified, being a 3.60% of pixels.

### 4.3. Summary

In this study, the results of serial and parallel versions of the KNN filtering algorithm for the classification of in-vivo brain tumor for hyperspectral images are introduced. In particular, the importance of reducing the area of the neighbors search in order to decrease the elaboration time is explained. In fact, the results prove that searching the neighbors of a pixel within a window instead of the entire image supposes a significant reduction of the computation time. It is important to notice that introducing a window (characterized by 14 rows with the reference pixel in the center) does not affect the result of the classification. For this reason, this version has been defined as the reference one (*WSize14*). Reducing the window size compared to the reference one, the time of the computation drastically decreases but the number of pixels that the algorithm misclassifies increases. At this point, it is important to select the best versions that have a good tradeoff between performance and number of misclassifications.

In the previous sections, the percentages and the number of different pixels between versions with different window sizes were analyzed. Concerning the implementations that exploit the Euclidean distance, Table 4 demonstrated that by using the window sizes *WSize12*, *WSize10* and *WSize8*, the number of different pixels was lower than those obtained using the *WSize6*, *WSize4* and *WSize2*, compared with the reference test (*WSize14*).

**Figure 10:**
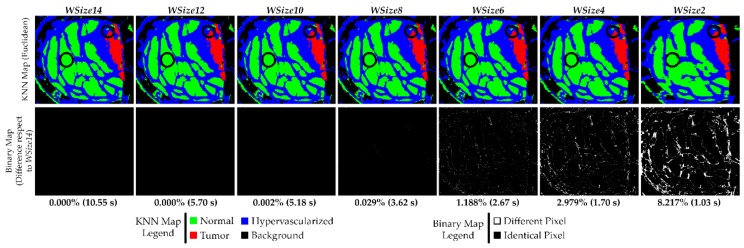
Results of the KNN filtering algorithm obtained from the P2C1 image using the Euclidean distance. The first row shows the filtered classification maps generated using different window sizes. The second row presents the binary maps where the pixel differences between the current generated map and the reference one (*WSize14*) are shown*.* In addition, the percentage of differences and the execution time results are detailed.

In Figure 10, the KNN filtered maps obtained from the P2C1 image and the binary maps are shown, where the differences between the evaluated window size version and the reference version are highlighted. Despite the differences between the first and the last three versions shown in Table 4, it is possible to see that the KNN filtered maps of the implementations for *WSize6* and *WSize8* do not present relevant dissimilarities. In fact, it is important to remember that the main goal of these maps is to delineate the tumor area, in order to provide the surgeons with a guidance tool during the tumor resection. In this context, it is clear that the number of different pixels in *WSize8* and *WSize6* versions are not so significant for the final application of the system, since the surgeon always resect a security margin around the tumor tissue.

In the previous paragraph, the data showed that the algorithm is able to correctly discriminate between tumor and healthy classes. This consideration can also be seen in the KNN filtered maps, where the area related to the tumor tissue (red) remains roughly the same in the implementations *WSize6*, *WSize8*, *WSize10* and *WSize12* compared to the *WSize14* one. Considering the *WSize4* and the *WSize2* versions, it is possible to appreciate that the margins of the tumor are not as evident and well defined as in the other images, confirming what has been said in the previous paragraphs while analyzing the classification results.

The second row of Figure 10 presents the binary maps that show the pixel differences between all the window size versions and the reference implementation (*WSize14*). In particular, by analyzing the binary maps of *WSize4* and *WSize2*, it is possible to identify several differences compared to *WSize14*. For this reason, these two versions should not be chosen for the final solution. However, in the binary maps of *WSize6* and *WSize8*, there are few differences, and they are barely appreciated analyzing the KNN filtered maps. It is important to remember that the suitable version for this application is the option that offers a good compromise between accurate classification and fast execution. Exploiting the GPU technology, the parallel version of the KNN algorithm with *WSize8* takes ~3.62 s to filter the P2C1 image, while the *WSize6* implementation is executed in ~2.67 s. For the biggest image of the database (P1C2), the *WSize6* implementation allows to save ~2 s when compared to the *WSize8* version. According to these results, the *WSize8* version has been selected as the best solution, giving priority to the classification accuracy but considering also a fast implementation. On the contrary, the *WSize6* implementation has been chosen as the fastest implementation with acceptable accuracy results.

**Figure 11:**
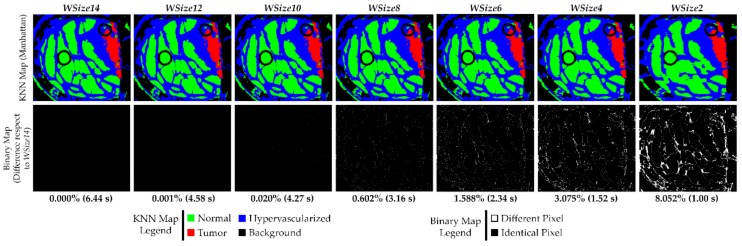
Results of the KNN filtering algorithm obtained from the P2C1 image using the Manhattan distance. The first row shows the filtered classification maps generated using different window sizes. The second row presents the binary maps where the pixel differences between the current generated map and the *WSize14*. In addition, the percentage of differences and the execution time results are detailed.

Similarly, the same evaluation can be done considering the implementations that exploit the Manhattan metric for the computation of the distances. Analyzing the computational times in the previous sections, it is evident that this metric leads to faster executions than the Euclidean distance. The first row of Figure 11 shows the KNN filtered maps of the P2C1 image using different window sizes and using the Manhattan distance. The second row presents the binary maps to evaluate the differences between the developed versions when compared to the *WSize14* implementation. According to the data shown in Table 7, in Figure 11 it is possible to see that the KNN filtered maps of the versions *WSize12* and *WSize10* are practically identical to the map obtained with *WSize14*. The number of different pixels is low enough to not perceive the differences between the classification maps. In Table 7, it is also evident that the number of different pixels from the *WSize8* to the *WSize2* implementations drastically increases. Concerning the KNN filtered maps of the *WSize4* and, in particular, the *WSize2* implementations, the differences are very clear since the margin of the tumor is not as well defined as in the other maps. Instead, in the filtered maps of the *WSize8* and *WSize6* implementations, the classification differences are not so evident, especially taking into account the tumor tissue area. The differences between all the versions compared to the *WSize14* implementation can be evaluated in the binary maps (Figure 11, second row). Even if the *WSize4* and *WSize2* are the fastest implementations, their binary maps clearly show that these two versions cannot be chosen because the amount of different pixels compared to *WSize14* is too high. However, in the binary maps of *WSize12* and *WSize10*, it is evident that these implementations offer the highest accuracy but the slowest execution times. Finally, concerning the *WSize8* and *WSize6* implementations, it is possible to determine that the *WSize8* version has the highest accuracy but the execution time is slower than the *WSize6* version (the former exhibits 3.16 s and the latter 2.34 s). Also in this case, the best solution is chosen on the basis of the degree of accuracy and the time constraints that the application requires.

At this point, the best solutions selected between the Manhattan versions (*WSize8* and *WSize6*) have to be compared with the reference test *WSize14* that exploits the Euclidean distance. The original algorithm is characterized by the use of the Euclidean metric in the neighbors search within the entire image. Since the *WSize14* Euclidean implementation does not have any differences in the classification results compared to the original version, the results of the Manhattan best solutions have to be compared with the reference results.

**Figure 12:**
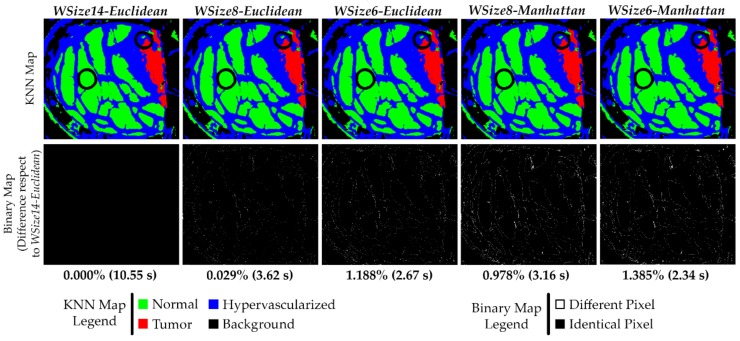
Results comparison of the KNN filtered maps from the P2C1 image using both the Manhattan and Euclidean distances. The first row shows the filtered classification maps generated using different window sizes and distance metrics. The second row presents the binary maps where the pixel differences between the current generated map and the reference one (*WSize14-Euclidean*) are shown. In addition, the percentage of differences and the execution time results are detailed.

By analyzing the results comparison shown in Figure 12, it is possible to see that all these versions have a reduced percentage of different pixels compared to the *WSize14-Euclidean* implementation. In all the obtained KNN filtered maps, the boundaries of the tumor area are accurately defined.

**Figure 13:**
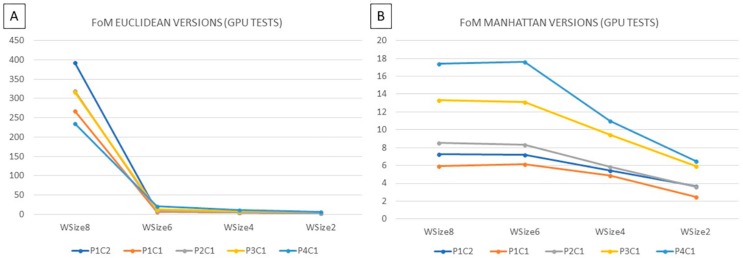
Figure of metric computed comparing (**A**) the Euclidean versions *WSize8*, *WSize6*, *WSize4* and *WSize2* with the reference *WSize14-Euclidean*, (**B**) the Manhattan versions *WSize8*, *WSize6*, *WSize4* and *WSize2* with the reference *WSize14-Euclidean*.

The solutions where the results are more similar to the reference implementation are the *WSize8-Euclidean* and *WSize8-Manhattan* versions, which differ 0.029% and 0.978% respectively, compared to the *WSize14-Euclidean* reference. The versions characterized by a window with 6 rows are less accurate than the previous ones, but they are faster. Concerning the computational times, the parallel execution of the reference solution is executed in ~10.55 s, while the *WSize8-Euclidean* and *WSize8-Manhattan* versions are executed in 3.62 and 3.16 s, respectively. The *WSize6-Euclidean* and *WSize6-Manhattan* implementations require 2.67 and 2.34 s, respectively. Finally, a figure of merit (*FoM* in Equation (5)), which relates the execution time (*t*) and the classification results (*err*), was considered to select the best solution that offers the highest value. The version *WSize8-Euclidean* is chosen as the best solution since it presents the highest value of *FoM* (Figure 13). To the best of our knowledge, the state of the art does not provide implementations of the KNN filtering algorithm that could be a touchstone for a fair comparison with the presented work.

(5) $$FoM=\frac{1}{\left(t\times err\right)}\;$$

## 5. Conclusions

This work presents the development of a parallel version of the KNN filtering algorithm exploiting the NVIDIA Tesla K40 board. The goal of the implementation was to reduce the execution time of the KNN filter to reach real-time constraints, which is mandatory considering the final application of the system. This application is related with the detection and identification of in-vivo brain tumor boundaries during neurosurgical operations by using hyperspectral images. For every pixel of the image, the parallel version of the algorithm computes each phase of the algorithm simultaneously. A first optimization was to introduce a *search window* in the K nearest neighbors search step, which is the most time-consuming part of the algorithm. The selection of the neighbors within a region close enough to the pixel, instead of the entire image, allows to significantly reduce the computational time of the algorithm. Furthermore, variations of the window sizes have been explored in order to evaluate the accuracy of the results and a possible reduction of the computational time. All the variations were considered exploiting both the Euclidean and the Manhattan metrics for the distance computation. The results obtained in this analysis show that, for the proposed final application, the implementation characterized by a search window of 8 rows using Euclidean distance is the best solution. This version performs the classification of the considered images in less than 6 s, with speedups up to 102.5× and 4317.9× compared with the *WSize14-Euclidean* and the *entire image* versions, respectively. Further developments must be carried out to integrate this parallel version of the KNN filtering algorithm with the other parts of the brain cancer detection algorithm (i.e., SVM classifier and PCA) in a single system capable of computing the classification maps of the hyperspectral brain cancer images in surgical-time to assist neurosurgeons during the resection of the tumor tissues.

## Figures and Tables

**Table 1:** HS brain cancer image database.

Image ID	#Pixels	Dimensions (Width × Height × Bands)
P1C1	251,532	548 × 459 × 128
P1C2	264,408	552 × 479 × 128
P2C1	219,232	496 × 442 × 128
P3C1	185,368	493 × 376 × 128
P4C1	124,691	329 × 379 × 128

**Table 2:** Execution times of the serial code considering as search space both the entire image (*EI* Time) and a window with 14 rows (*WSize14* Time).

Image ID	#Pixels	*EI* Time [s]	*WSize14* Time [s]	Speedup	Min *WSize14* [#Pixels]	Max *WSize14* [#Pixels]
P1C1	251,532	17,173.74	503.89	34.08×	3836	7672
P1C2	264,408	19,135.58	509.16	37.58×	3864	7728
P2C1	219,232	15,630.77	374.67	41.72×	3472	6944
P3C1	185,368	9788.58	322.86	30.32×	3451	6902
P4C1	124,691	4015.89	139.30	28.83×	2303	4606

**Table 3:** Execution time results of the serial and parallel implementations using the Euclidean distance employing different window sizes.

Image ID	Processing Type	Processing Time [s]						
		*WSize14*	*WSize12*	*WSize10*	*WSize8*	*WSize6*	*WSize4*	*WSize2*
P1C1	Serial	503.89	406.32	383.71	262.00	221.71	118.25	59.08
	CUDA	12.83	11.49	6.23	5.52	3.85	2.29	1.22
	Speedup	39.25×	35.33×	61.59×	47.42×	57.52×	51.44×	48.10×
P1C2	Serial	509.16	424.22	408.52	276.06	235.76	125.34	62.36
	CUDA	13.53	12.09	6.47	5.73	3.99	2.39	1.26
	Speedup	37.62×	35.08×	63.07×	48.11×	59.04×	52.31×	49.15×
P2C1	Serial	374.67	315.73	302.54	239.58	151.23	95.02	47.06
	CUDA	10.55	5.70	5.18	3.62	2.67	1.70	1.03
	Speedup	35.51×	55.39×	58.30×	66.18×	56.58×	55.76×	45.62×
P3C1	Serial	322.86	263.40	254.30	202.56	122.16	78.47	39.97
	CUDA	9.00	4.92	4.45	3.15	2.30	1.51	0.92
	Speedup	35.85×	53.46×	57.06×	64.17×	52.92×	51.80×	43.16×
P4C1	Serial	139.30	118.94	115.07	90.81	55.29	35.84	18.11
	CUDA	3.21	2.34	2.16	1.63	1.12	0.83	0.60
	Speedup	43.38×	50.63×	53.23×	55.61×	49.01×	42.82×	30.13×

**Table 4:** Number of pixels with different classification result using the Euclidean distance between the different computed windows sizes and the reference one (*WSize14*).

Image ID	#Pixels	#Different Pixels (% of Difference) Compared to the Reference (*WSize14*)					
		*WSize12*	*WSize10*	*WSize8*	*WSize6*	*WSize4*	*WSize2*
P1C1	251,532	0 (0.000%)	0 (0.000%)	65 (0.026%)	3672 (1.460%)	9096 (3.616%)	20,476 (8.141%)
P1C2	264,408	0 (0.000%)	1 (0.000%)	60 (0.023%)	2845 (1.076%)	7705 (2.914%)	22,054 (8.341%)
P2C1	219,232	0 (0.000%)	4 (0.002%)	65 (0.030%)	2606 (1.189%)	6532 (2.979%)	18,015 (8.217%)
P3C1	185,368	0 (0.000%)	1 (0.000%)	49 (0.026%)	2273 (1.226%)	5604 (3.023%)	13,981 (7.542%)
P4C1	124,691	3 (0.002%)	7 (0.005%)	71 (0.057%)	1498 (1.201%)	3733 (2.993%)	10,089 (8.091%)

**Table 5:** Comparison of the execution time of the serial versions obtained employing the Euclidean and Manhattan distances with the entire image (*EI*) and the *WSize14*. The table also presents the classification differences between the Euclidean and Manhattan implementations.

Distance Type	P1C1		P1C2		P2C1		P3C1		P4C1	
	*EI*	*WSize14*	*EI*	*WSize14*	*EI*	*WSize14*	*EI*	*WSize14*	*EI*	*WSize14*
Euclidean [s]	17,173.75	503.89	19,135.58	509.17	15,630.77	374.67	9788.58	322.87	4015.89	139.30
Mahattan [s]	7222.13	190.02	7683.44	202.42	4735.87	146.93	3382.84	121.37	1807.91	55.91
Speedup	2.38×	2.65×	2.49×	2.51×	3.3×	2.55×	2.89×	2.66×	2.22×	2.49×
Difference	1.33%		0.99%		1.03%		1.10%		1.06%	

**Table 6:** Execution time results of the serial and parallel implementations using the Manhattan distance and using different window sizes.

Image ID	Processing Type	Processing Time [s]						
		*WSize14*	*WSize12*	*WSize10*	*WSize8*	*WSize6*	*WSize4*	*WSize2*
P1C1	Serial	190.02	192.19	158.15	129.54	81.61	54.14	28.70
	CUDA	7.63	7.07	5.04	4.62	3.38	2.04	1.18
	Speedup	24.90×	27.15×	31.34×	27.99×	24.09×	26.47×	24.13×
P1C2	Serial	202.41	204.65	169.23	138.12	84.98	57.51	29.75
	CUDA	8.01	7.40	5.21	4.84	3.51	2.09	1.22
	Speedup	25.26×	27.64×	32.48×	28.52×	24.20×	27.45×	24.28×
P2C1	Serial	146.92	152.20	125.60	102.94	63.90	42.57	21.81
	CUDA	6.44	4.58	4.27	3.16	2.34	1.52	1.00
	Speedup	22.81×	33.20×	29.35×	32.50×	27.25×	27.86×	21.66×
P3C1	Serial	121.37	126.98	104.79	86.83	55.09	36.24	18.54
	CUDA	5.57	4.03	3.76	2.79	2.04	1.37	0.90
	Speedup	21.75×	31.47×	27.86×	31.11×	26.88×	26.42×	20.54×
P4C1	Serial	55.91	58.06	42.62	39.42	24.39	16.62	8.66
	CUDA	2.81	2.12	1.98	1.49	1.04	0.80	0.60
	Speedup	19.87×	27.27×	21.46×	26.46×	23.31×	20.59×	14.42×

**Table 7:** Number of pixels with different classification results using the Manhattan distance between the computed window sizes and the *WSize14* Manhattan.

Image ID	#Pixels	#Different Pixels (% of Difference) Compared to the *WSize14* (*Manhattan*) Version					
		*WSize12*	*WSize10*	*WSize8*	*WSize6*	*WSize4*	*WSize2*
P1C1	251,532	2 (0.001%)	65 (0.026%)	1832 (0.728%)	4507 (1.792%)	8889 (3.534%)	19,783 (7.865%)
P1C2	264,408	3 (0.001%)	56 (0.021%)	1483 (0.561%)	3891 (1.471%)	7839 (2.965%)	21,714 (8.812%)
P2C1	219,232	3 (0.001%)	44 (0.020%)	1320 (0.602%)	3483 (1.589%)	6743 (3.075%)	17,653 (8.052%)
P3C1	185,368	2 (0.001%)	48 (0.026%)	1033 (0.557%)	2825 (1.524%)	5474 (2.953%)	13,436 (7.248%)
P4C1	124,691	2 (0.001%)	35 (0.028%)	699 (0.560%)	2014 (1.615%)	3831 (3.072%)	9613 (7.709%)
